# An Atypical Case of Head Tremor and Extensive White Matter in an Adult Female Caused by 3-Hydroxy-3-methylglutaryl-CoA Lyase Deficiency

**DOI:** 10.3390/diagnostics11091561

**Published:** 2021-08-28

**Authors:** Nassim Boutouchent, Julie Bourilhon, Bénédicte Sudrié-Arnaud, Antoine Bonnevalle, Lucie Guyant-Maréchal, Cécile Acquaviva, Loréna Dujardin-Ippolito, Soumeya Bekri, Ivana Dabaj, Abdellah Tebani

**Affiliations:** 1 Normandie University, UNIROUEN, INSERM U1245, CHU Rouen, Department of Metabolic Biochemistry, 76000 Rouen, France; Nassim.Boutouchent@chu-rouen.fr (N.B.); b.sudrie-Arnaud@chu-rouen.fr (B.S.-A.); L.Dujardin-Ippolito@chu-rouen.fr (L.D.-I.); soumeya.bekri@chu-rouen.fr (S.B.); 2 Rouen University Hospital, CHU de Rouen, Department of Neurology, 76000 Rouen, France; Julie.Bourilhon@chu-rouen.fr (J.B.); Antoine.Bonnevalle@chu-rouen.fr (A.B.); 3 Department of Neurophysiology, Rouen University Hospital, 76000 Rouen, France; Lucie.Guyant@chu-rouen.fr; 4 Department of Biochemistry and Molecular Biology, Inborn Errors of Metabolism, Center of Biology and Pathology Est, CHU Lyon, 69310 Bron, France; cecile.acquaviva-bourdain@chu-lyon.fr; 5 Normandie University, UNIROUEN, INSERM U1245, CHU Rouen, Department of Neonatal Pediatrics, Intensive Care and Neuropediatrics, 76000 Rouen, France; ivana.dabaj@chu-rouen.fr

**Keywords:** HMGLD, *HMGCL*, HMG-CoA lyase deficiency, NGS, inherited metabolic diseases

## Abstract

3-Hydroxy-3-methylglutaryl-CoA (HMG-CoA) Lyase deficiency (HMGLD) (OMIM 246450) is an autosomal recessive genetic disorder caused by homozygous or compound heterozygous variants in the *HMGCL* gene located on 1p36.11. Clinically, this disorder is characterized by a life-threatening metabolic intoxication with a presentation including severe hypoglycemia without ketosis, metabolic acidosis, hyper-ammoniemia, hepatomegaly and a coma. HMGLD clinical onset is within the first few months of life after a symptomatic free period. In nonacute periods, the treatment is based on a protein- and fat-restricted diet. L-carnitine supplementation is recommended. A late onset presentation has been described in very few cases, and only two adult cases have been reported. The present work aims to describe an incidental discovery of an HMGLD case in a 54-year-old patient and reports a comprehensive review of clinical and biological features in adult patients to raise awareness about the late-onset presentation of this disease.

## 1. Introduction

**Figure 1:**
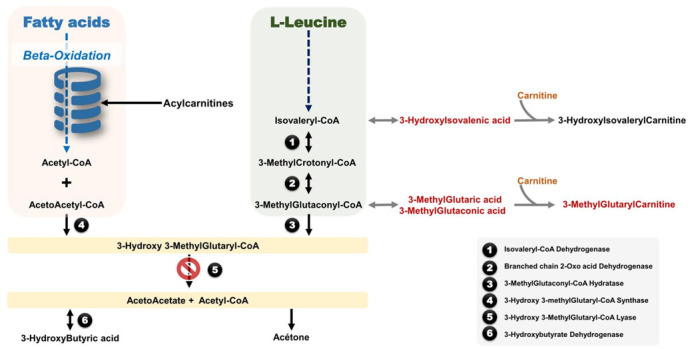
Overview of the involved metabolic pathways in the 3-Hydroxy-3-methylglutaryl-CoA Lyase (HMG-CoA Lyase) metabolism.

The 3-Hydroxy-3-methylglutaryl-CoA Lyase (HMG-CoA Lyase) enzyme is involved in both L-Leucine catabolism and ketone bodies anabolism (Figure 1). HMG-CoA Lyase deficiency (HMGLD) (OMIM 246450) is an autosomal recessive genetic disorder caused by homozygous or compound heterozygous pathogenic variants in the *HMGCL* gene located on 1p36.11. Clinically, this disorder is characterized by a life-threatening metabolic intoxication with a presentation including severe hypoglycemia without ketosis, metabolic acidosis, hyperammonemia, hepatomegaly and, invariably, a coma state triggered by an energy crisis such as an infection, vaccination or low dietary intake [1,2]. HMGLD clinical onset is within the first few months of life after a symptomatic free period. Indeed, the metabolic crisis occurs in ~70% of cases before the age of 1 year [1]. In addition, several neurological manifestations such as epilepsy, lethargy and irritability as well as non-neurological manifestations including hepatomegaly, anaemia and eating difficulties have been reported in older children [2]. The diagnosis is based on (i) a urine organic acids analysis with a typical profile including high levels of 3-Hydroxy-3-MethylGlutaric, 3-MethylGlutaric, 3-MethylGlutaconic and 3-HydroxyIsovaleric acids [3], and (ii) an acylcarnitine profile revealing a high level of 3-hydroxy-isovalerylcarnitine with a decreased free carnitine concentration [3] (Figure 1). The molecular study of the *HMGCL* gene enables one to confirm the diagnosis. Fifty-one pathogenic variants have been reported in the Public Human Gene Database (HGMD—http://www.hgmd.cf.ac.uk/ac/index.php—1 May 2021).

Hypoglycemia and ketone bodies shortage may underlie the pathophysiological mechanisms of this disease since acetoacetate and 3-hydroxybutyrate, named ketone bodies, are used as primary substrates for energy supply by several organs such as the central nervous system and the cardiac and skeletal muscles. Moreover, a redox balance disruption through the accumulation of toxic metabolites and a decreased carnitine level may contribute to the metabolic crisis [4,5]. This hypothesis is supported by an in vivo animal study suggesting that the long-term accumulation of key metabolites in HMGCLD induces an increase in the level of oxidative stress correlated with brain toxicity. These data could possibly account for the neurological presentation of this disease [6].

In nonacute periods, the treatment is based on a protein- and fat-restricted diet, and L-carnitine supplementation is recommended. The latter has a major role in organic acids’ removal and their urinary elimination, and thus it acts as an antioxidant agent by reducing metabolites’ accumulation [2,7]. Besides, a recent report highlights the efficacy of sodium DL-3-hydroxybutyrate as an adjuvant treatment for HMG-CoA lyase [8].

A late-onset presentation has been described in very few cases. Some cases have been reported in infancy and late childhood [9,10], while, to our knowledge, only two adult cases have been described [11,12]. The present work aims to describe an incidental discovery of an HMGLD case in a 54-year-old patient and reports a comprehensive review of clinical and biological features in adult patients in order to get more insight into the late-onset presentation of this disease.

## 2. Patient and Methods

### 2.1. Case Description

A fifty-four year old female patient presented with cervical dystonia and head tremor with lateral movement (no-no) from age 48. The past medical history includes an unexplained coma for three days at the age of three years, followed by childhood epilepsy. Seizures were controlled by phenobarbital given until the age of 14 years. She also suffered from substituted hypothyroidism, hypercholesterolemia and hypertension (regulated by appropriate therapy). She does not have any learning difficulties or developmental delay. She was born from non-consanguineous parents. The family history reveals that her older sister had epilepsy and severe mental retardation. The clinical examination confirmed a right laterocollis associated with a no-no head tremor. No cerebellar syndrome has been noticed. In addition, routine biological tests have shown a moderate hyperammonaemia of 69 µmol/L (normal range: 11–35 µmol/L), absence of acid-base disorders and a normal glycemia of 5.1 mmol/L (reference range: 4–6 mmol/L). Of note, the analysis of cerebrospinal fluid showed a moderate hyperproteinorachia (0.62 g/L; N < 0.4).

### 2.2. Brain Magnetic Resonance Imaging (MRI)

A cerebral MRI was performed using T2 weighting Fluid Attenuated Inversion Recovery (FLAIR) sequences associated with a long echo time (TE) [13]. This technique makes the CSF signal null and improves the detection of brain parenchyma lesions [14], making it very useful for cerebral imaging.

### 2.3. Biochemical Investigations

For urinary organic acids, urines samples were subjected to derivatization with N,O-bis(trimethylsilyl)trifluroacetamide and trimethylchlorosilane. Derivatized samples were injected into a Shimadzu QP-2010 Plus GC-MS operating in split mode. The metabolites were analyzed as trimethylsilyl compounds. Heptadecanoic acid was used as an internal standard. A Blood Acylcarnitine Profile on a dried blood spot was generated using butylation derivatization (ChromSystems^®^, Munich, Germany) and measured by MS/MS on a 4000 QTRAP (Sciex^®^, Concord, ON, Canada). The acylcarnitine butylated esters were acquired by precursor ion scanning of 85 *m/z* in positive ion mode.

### 2.4. Molecular Analysis

Genomic DNAs were tested by next-generation sequencing (NGS) using a custom design based on a SeqCap EZ Solution-Based Enrichment strategy (Roche NimbleGen, Madison, WI, USA). Targeted sequencing capture probes were custom designed by Roche NimbleGen. Targeted regions include exons and exon-intron boundaries (exon ± 25 nt), 5′- and 3′-UTR regions of genes involved in inborn metabolic diseases, including HMGCL gene. Sequencing was performed on a NextSeq500 sequencer using the NextSeq500 Mid Output Kit v2 (300 cycles) chemistry (Illumina, San Diego, CA, USA). Bioinformatic analyses were performed using a homemade pipeline according to the GATK Best Practices recommendations (PMID: 25431634). Putative identified pathogenic variants were verified by conventional dideoxy sequencing using the BigDye Terminator v.3.1 Cycle Sequencing Kit (Life Technologies, Carlsbad, CA, USA). Variants were named according to HGVS recommendations, using the NM_000191 sequence. Analyses of the variants were performed with Alamut v2.11 software (Interactive Biosoftware, Rouen, France), and scoring of 5′ and 3′ splice sites was performed using Neural Network Splice Prediction (NNSplice), MaxEntScan, Splice site Finder Like, GeneSplicer, Human Splicing Finder (HSF).

## 3. Results

### 3.1. Brain MRI

**Figure 2:**
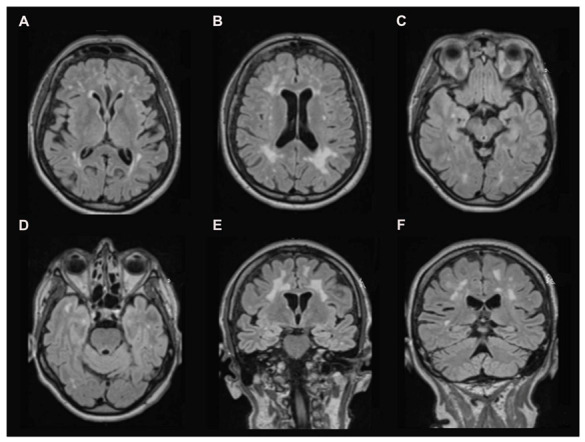
(**A**–**C**) Axial T2-weighted magnetic resonance images show a high signal intensity in patchy, confluent periventricular and subcortical areas of the white matter with a clear predominance in supratentorial areas. The basal ganglia and brainstem did not show any abnormalities. (**D**) Axial T2-weighted magnetic resonance images and (**E**) coronal T2-weighted magnetic resonance images show a slight temporal lobe involvement. (**F**) Coronal T2-weighted magnetic resonance images show no cerebellar abnormalities.

The brain MRI revealed severe symmetrical supratentorial with confluent periventricular and subcortical white matter hyperintensities on T2-FLAIR (Figure 2A–C) weighted images without abnormal contrast enhancement. Interestingly, the temporal lobes and u-fibers are minimally involved (Figure 2D–F). The basal ganglia, brainstem and cerebellum are spared (Figure 2).

### 3.2. Biochemical Investigations

HMGLD diagnosis has been achieved by the detection of an elevated urinary concentration of 3-Hydroxy-3-MethylGlutaric, 3-MethylGlutaric, 3-MethylGlutaconic and 3-HydroxyIsovaleric acids. The analysis of acylcarnitines revealed an increased level of 3-HydroxyIsovalerylcarnitine, which supports the diagnosis (Table 1). Interestingly, a normal range of carnitine concentration has been retrieved without any therapeutic supplementation.

### 3.3. Molecular Analysis

*HMGCL* gene sequencing enabled the characterization of two pathogenic variants: NM_000191.2:c.144G>C-p.Lys48Asn, a variant previously described [15], and c.60+1G>C-p ? The latter has never been described and is predicted to abolish the splicing donor site and to cause exon 1 skipping. The allelic segregation was not performed as the DNA samples from the parents were not available for us.

## 4. Discussion

HMGCLD is a rare inborn error of ketone bodies synthesis and leucine degradation. It has a typical onset in the first few months of life with an initial presentation mimicking Reye syndrome, including recurrent vomiting, seizures and impaired vigilance [1]. The long-term outcome in older children is characterized by neurological complications such as epileptic seizures, muscular hypotonia and tremor associated with marked white matter lesions in the brain MRI [2]. The clinical expression of inborn errors of metabolism (IEM) is now viewed as a severity continuum with a wide clinical spectrum spanning from a severe presentation in the prenatal or neonatal period to moderate or even asymptomatic adult forms. The adult presentation of this IEM is underdiagnosed, and therefore the awareness of adult physicians is still to be improved. Here, we report an adult presentation of HMGCLD aiming to widen the clinical knowledge of this rare disease and raise awareness among the different stakeholders involved in the management of IEM.

To the best of our knowledge, only two adult presentations have been reported so far (Supplementary Table S1). Reimão et al. described a 29-year-old man with no prior medical history who presented with a sudden-onset coma, profound hypoglycemia, hyperammonaemia and metabolic acidosis without ketosis. The patient died with multi-organic failure within five days [12]. Bischof et al. reported the case of a 36-year-old woman with an acute episode of hypoglycaemia, hyperproteinorachia (0.73 g/L) and generalized seizures [11]. Her medical history is marked by recurrent episodes of somnolence and hypoglycemia starting from the neonatal period. During childhood, she presented seizures and developmental delay. This patient had carnitine supplementation, and some clinical features improved markedly [11]. In contrast, our patient’s adult onset was not revealed by a metabolic decompensation, and the neurological impairment was in the forefront of the clinical picture. It is worth noting that our patient presented with cervical dystonia and head tremor. Regarding the head tremor, this feature has been observed in 9% of the reported cases [2], while the cervical dystonia has not been reported yet in HMGCLD and could thus be incidental. Indeed, considering the age of our patient, idiopathic dystonia cannot be excluded.

Biochemical investigations retrieved typical urine organic acids and blood acylcarnitine profiles. Molecular analysis confirmed the diagnosis by the identification of two heterozygous pathogenic variants (NM_000191.2:c.144G>C-p.Lys48Asn and c.60+1G>C-p ?).

The MRI findings in our case are very close to those described by Bischof et al. [11] with a supratentorial confluent leukoencephalopathy sparing basal ganglia. The U-fibers are slightly involved in our patient, while they were spared in the patient described by Bischof et al. [11]. The patient described by Reimão et al. [12] presented with a prominent clinical expression and a more severe leukoencephalopathy and U-fibers’ involvement. Interestingly, the brainstem and cerebellum were normal too. Temporal involvement was not reported in either of the reported cases. It is interesting to note the absence of the brainstem involvement and the basal ganglia, which are usually affected in hypertensive leukoencephalopathy. Likewise, the predominance of the lesions in supratentorial areas with a relative sparing of the temporal lobes is not in favor of CADASIL disease [16].

## 5. Conclusions

Although HMGLD is quite a rare condition, it should be considered in cases with extensive white matter lesions with relatively spared brainstem and temporal lobes. The integrative interpretation of imaging, biochemical and molecular findings enable one to reach the diagnosis of this treatable condition.

## Figures and Tables

**Table 1:** Overview of the biochemical investigation results.

Investigation	Metabolite	Concentration	Reference Range
Urinary Organic Acids(mmol/mol of Creatinine)	3-Hydroxy-3-MethylGlutaric acid	304	<200
	3-hydroxyIsovaleric acid	143	<50
	3-methylGlutaric acid	54	<5
	3-methylGlutaconic acid	399	<25
Blood Carnitines(µmol/L)	Carnitine	20	15–35
	3-hydroxyIsovalerylcarnitine	3.73	<0.38

## Data Availability

All data that support the findings are included in the manuscript and in the supplemental data.

## Supplementary Materials

The following are available online at https://www.mdpi.com/article/10.3390/diagnostics11091561/s1, Table S1: Clinical data overview.
